# Reproduction and Development in Fish: Solving Bottlenecks in Modern Aquaculture

**DOI:** 10.3390/ani15020145

**Published:** 2025-01-09

**Authors:** Renato Massaaki Honji, Paulo Henrique de Mello, Bruno Cavalheiro Araújo

**Affiliations:** 1 Laboratório de Aquicultura e Ecofisiologia Marinha (LAQUEFIM), Departamento de Fisiologia, Instituto de Biociências, Universidade de São Paulo (IB/USP), Rua do Matão, Travessa 14, nº 321, São Paulo 05508-090, SP, Brazil; 2 Beacon Development, King Abdullah University of Science and Technology, Thuwal 23955-6900, Saudi Arabia; paulo.demello@kaust.edu.sa; 3 Laboratório de Fisiologia e Nutrição de Organismos Aquáticos (LAFINUTRI), Núcleo Integrado de Biotecnologia, Universidade de Mogi das Cruzes, Avenida Dr. Cândido Xavier de Almeida e Souza, nº 200, Mogi das Cruzes 08701-970, SP, Brazil; brubiol1@gmail.com

## 1. Introduction

The aquaculture industry is home to the fastest-growing activity in the agriculture sector worldwide and is one of the leading sectors in global food production [1,2,3]. Global aquaculture has proven to be an alternative source of food production due to population growth, exceeding 8 billion people in 2024, thus forcing the population to seek alternative food resources to solve problems related to hunger, therefore, highlighting the importance of both freshwater and marine aquaculture [2,4]. On the other hand, the sustainable growth of this important activity depends on controlling two complex and crucial areas: reproduction and larval rearing [2,3,5]. These processes are central to the economic feasibility and sustainability of the activity [2,3,4,5], but they face different challenges, including environmental variables and biological mechanisms [4,6].

The present Special Issue “Reproduction and Development in Fish: Solving Bottlenecks in Modern Aquaculture”, strengthens cutting-edge research addressing these complex challenges. This Special Issue presents up-to-date regular research and reviews that address the complex challenges in fish reproduction and larval development, crucial areas for sustaining and advancing modern aquaculture. The insights shown are not just incremental improvements; they are essential advances that promise to improve the aquaculture industry and promote its sustainability and growth.

### 1.1. Complexities of Fish Reproduction

Reproductive efficiency in aquaculture is one of the main bottlenecks for this industry’s economic viability and sustainability [7,8]. On the other hand, achieving optimal reproductive results in fish remains fraught with challenges that require different and specific hormonal therapies (different delivery systems to promote final oocyte maturation, ovulation, and spawning/spermiation) and/or modulation of the environmental factors (changes in photoperiod, temperature, salinity, among other physical and chemical factors), or even a combination of both hormonal therapies and the modulation of the environmental factors. All these interventions must be science-based. Currently, research is deepening the cutting-edge reproductive technologies, including the strategic use of hormone therapies and adequate broodstock nutrition to promote the production of viable gametes and favorable spawning/spermiation conditions, as observed in different teleost species [7,9,10,11,12]. These advances are necessary to maximize gamete production and improve quality, ensuring the continued proliferation of high-value species [5,7,13,14].

When in captivity, fish species exhibit some level of reproductive dysfunction as reported by many farmers on a commercial scale. Females often fail to reach final oocyte maturation, and ovulation and spawning do not occur, while males normally produce low amounts of sperm, often followed by low-quality sperm, which can lead to underdeveloped growth and reduced overall production efficiency [15,16,17]. The innovative strategies highlighted in this Special Issue, such as hormonal manipulation, immune, and nutrition strategies, offer promising alternatives for gamete maturation to improve animal growth in a healthier way. These methodologies are not merely theoretical; they are actionable practices with the potential to improve fish farming protocols [5,12].

Additionally, broodstock nutrition has also been addressed in this Special Issue, specifically related to the replacement and/or incorporation of important lipids, specific fatty acids (docosahexaenoic acid—DHA and eicosapentaenoic acid—EPA), and vitamins, all focused on reproductive success and animal growth. Emerging research highlights the importance of adjusted feeding regimens that not only improve gamete quality but also increase offspring viability. By optimizing broodstock nutrition, fish farmers can significantly increase reproductive output and the long-term sustainability of their operations, as described in several studies [7,8,12].

### 1.2. Advances in Fish Development

The initial developmental stages of fish are characterized by high sensitivity to adverse environmental conditions and nutritional inputs offered [18,19]. Any nutritional deficiency during these stages can result in a cascade of negative effects, i.e., altering growth, health, survival, and consequently production and profitability [20]. This Special Issue also highlights innovative tools that offer new insights into the larval development of species in all sectors of the aquaculture industry, including ornamental aquaculture. These tools are advancing our understanding of developmental biology and allowing the precise manipulation of growth parameters, ultimately leading to more robust and resilient aquaculture species [21,22]. Furthermore, it is important to mention that climate change [23,24] also influences all these processes in aquaculture.

Manipulation of environmental variables such as temperature, salinity, and photoperiod has long been recognized as a powerful lever to control fish development. Many studies explore the latest innovations in environmental modulation, demonstrating how these techniques can be adjusted to optimize growth rates and ensure the timely maturation of fish targeting the market demands [19,25]. The potential of these approaches to increase production efficiency while maintaining high animal welfare standards cannot be overstated. However, for many species with potential for aquaculture, basic studies aiming to understand embryonic and larval development are scarce. This topic is also of utmost importance, as it opens new pathways for the conservation of species and the diversification of the aquaculture industry [21].

### 1.3. The Imperative of Diversifying Aquaculture Species

As the aquaculture industry expands, there is a growing need to diversify the species farmed. Diversification is therefore critical to reducing the industry’s reliance on a limited number of species, thereby increasing its resilience to atypical environmental conditions and market fluctuations. In addition to the common species used in production, this Special Issue also addresses the unique challenges associated with reproduction, nutrition, and embryonic and larval development of emerging species, or new species with great potential for aquaculture, many of which are being domesticated for the first time. Successful integration of these species into rearing systems still depends on our ability to develop and adjust protocols for rearing, embryonic and larval development, and growth of these species [26].

Species diversification in aquaculture is not merely a response to high market demands but is a strategic necessity for global ecological and economic sustainability. The introduction of new species requires basic and advanced research to ensure that their reproductive, nutritional, and developmental processes can be effectively managed without compromising the environmental integrity or welfare of these species [26,27,28]. The contributions in this Special Issue provide valuable insights to achieve these goals, offering a guide for the sustainable expansion and diversification of global aquaculture.

### 1.4. A Call to Action for Future Research and Industry Innovation

Fish reproduction and development challenges are among the most critical bottlenecks facing the future of aquaculture. However, the papers published in this Special Issue demonstrate that these challenges are not impossible. Through interdisciplinary collaboration and the relentless pursuit of innovation, the aquaculture industry is well-positioned to overcome these barriers and achieve new levels of sustainability and productivity [7,14]. Furthermore, this Special Issue serves as a reference for current scientific understanding and a clear call for continued basic and advanced research. The tools, techniques, and insights presented here are transformative solutions that will shape the future of aquaculture [29]. As we move forward, it is critical that the industry embraces these innovations and integrates them into practice to ensure that aquaculture remains a viable, sustainable, and productive component of global food systems [1,2].

In conclusion, this Special Issue entitled “Reproduction and Development in Fish: Solving Bottlenecks in Modern Aquaculture” represents a significant leap forward in managing the reproduction and development of aquaculture species. The presented research not only highlights pivotal advancements but also underscores the rapid growth and dynamism in research and development efforts that are redefining industry standards and setting the stage for a more sustainable and resilient future in aquaculture. This issue, alongside other Special Issues within *Animals* that delve into related themes ([30,31,32,33,34,35,36,37,38,39,40]; among others), serves as a testament to the remarkable progress being made in these areas. As the scholarly landscape continues to expand, it presents a complex terrain that demands a discerning approach. We encourage readers to critically evaluate the scientific integrity of each contribution while recognizing the inherent value of knowledge, regardless of its origin. Thus, the guest editors are honored to present this Special Issue, inviting you to engage with the transformative insights and pioneering innovations that will shape the future of fish reproduction and aquaculture.
